# Alkaline-Earth-Promoted CO Homologation and Reductive Catalysis

**DOI:** 10.1002/anie.201505851

**Published:** 2015-07-24

**Authors:** Mathew D Anker, Michael S Hill, John P Lowe, Mary F Mahon

**Affiliations:** Department of Chemistry University of Bath, Bath BA2 7AY (UK)

**Keywords:** carbon monoxide, catalysis, Fischer–Tropsch synthesis, magnesium, reduction

## Abstract

Reaction between a β-diketiminato magnesium hydride and carbon monoxide results in the isolation of a dimeric *cis*-enediolate species through the reductive coupling of two CO molecules. Under catalytic conditions with PhSiH_3_, an observable magnesium formyl species may be intercepted for the mild reductive cleavage of the CO triple bond.

The deoxygenative conversion of carbon monoxide to hydrocarbon fuels and lower oxygenates is typically achieved through heterogeneous catalytic methods.^1^ Although Fischer–Tropsch (F-T) chemistry and related processes have been successfully implemented for some eighty years to produce a Schultz–Flory distribution of hydrocarbons, only limited success has been achieved with well-defined homogeneous systems, typically at CO pressures in excess of 1000 atm.^2^ More recent activity has targeted the synthesis of putative F-T intermediates including, and most pertinent to the current work, enediolates such as the zirconium and cerium species **I**–**III**, which were prepared through treatment of the relevant metal hydride with CO.^3^ Our own research efforts have focused on the development of a homogeneous catalytic chemistry for complexes derived from the inexpensive and environmentally benign alkaline-earth elements,^4^ in particular magnesium and calcium; respectively the eighth and fifth most abundant elements in the lithosphere. In this contribution we demonstrate that similar CO homologation reactivity may be achieved through exposure of the β-diketiminato magnesium hydride (**IV**)^5^ to one atmosphere of CO and that this and other alkaline earth species enable the highly selective catalytic reduction of the carbon monoxide molecule under similarly mild reaction conditions.


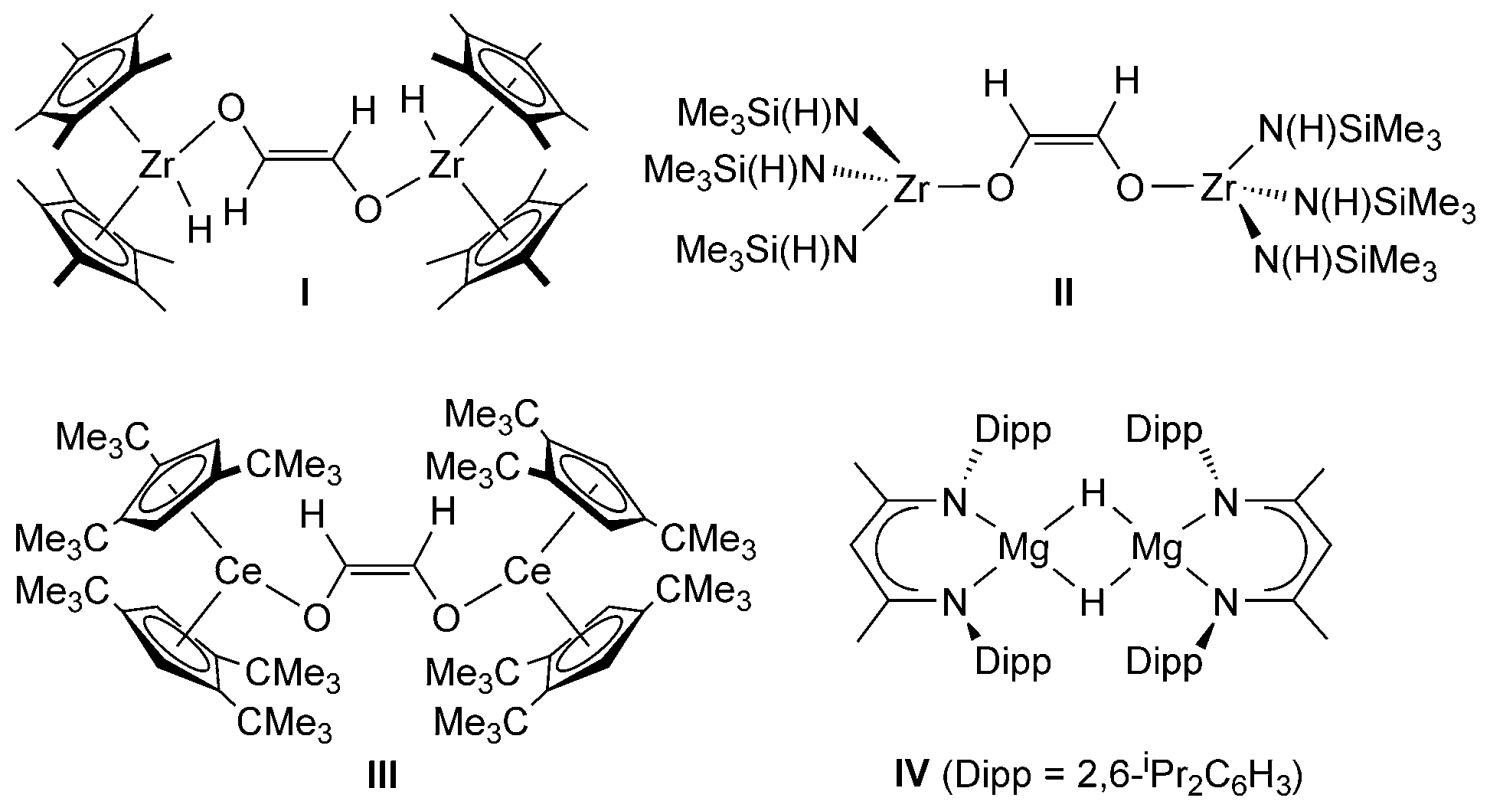


Compound **IV** reacted under one atmosphere of CO at room temperature to provide a single new species (**1**) characterized by the appearance of a singlet resonance at *δ* 5.53 ppm in the resultant ^1^H NMR spectrum with an accompanying vinylic carbon resonance at *δ* 131.7 ppm in the corresponding ^13^C{^1^H} NMR spectrum. Repetition of this reaction with ^13^CO provided ready access to the isotopomer (**1**-^13^C), which comprised diagnostic 10 line AA′XX′ patterns (^1^*J*_CH_=174.4, ^2^*J*_CH_=20.4, ^1^*J*_CC_=79.5, ^3^*J*_HH_=1.3 Hz) in both the ^1^H and gated ^13^C–^1^H NMR spectra (see Figures S1 and S2 in the Supporting Information). Replacement of the ^13^CO atmosphere of this sample with ^12^CO resulted in no simplification of the AA′XX′ spin system even at elevated temperatures indicating that the formation of compound **1** is irreversible. These spectral data are closely comparable to those reported for compounds **I**–**III** and are thus similarly indicative of the reductive coupling of two molecules of CO to form a dinuclear magnesium enediolate species [Eq. 1].^3^

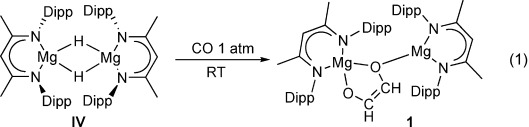
1

These deductions were confirmed through a subsequent X-ray structural analysis, the results of which are illustrated in Figure 1, performed on a selected single crystal of compound **1** grown from a saturated *n*-pentane solution. While the C30–C31 distance [1.327(3) Å] is clearly indicative of the formation of a C=C double bond and a *cis*-enediolate similar to that observed in both compounds **II** and **III**, the ligand does not display a directly analogous symmetrical bridging disposition. Rather, the planar *cis*-enediolate adopts an asymmetric bridging mode in which one four-coordinate pseudo-tetrahedral magnesium (Mg1) center is bound through chelation of both O1 [Mg1–O1 1.92(1) Å] and O2 [Mg1–O2 2.01(1) Å]. This latter bond is slightly elongated as O2 is also bound to the further trigonal Mg2 center [Mg2–O2 1.88(1) Å]. Variable-temperature ^1^H NMR studies performed in [D_8_]toluene did not show any change in the singlet resonance at *δ* 5.53 ppm down to the low-temperature limit of −90 °C (Figure S3). We, thus, suggest that the *cis-*enediolate chelate undergoes facile exchange between both Mg1 and Mg2 centers at a rate that is faster than the NMR time scale. The geometry and resultant asymmetry of compound **1** in the solid state is, therefore, suggested to be reflective of the lowest energy conformer and is not retained in solution.

**Figure 1:**
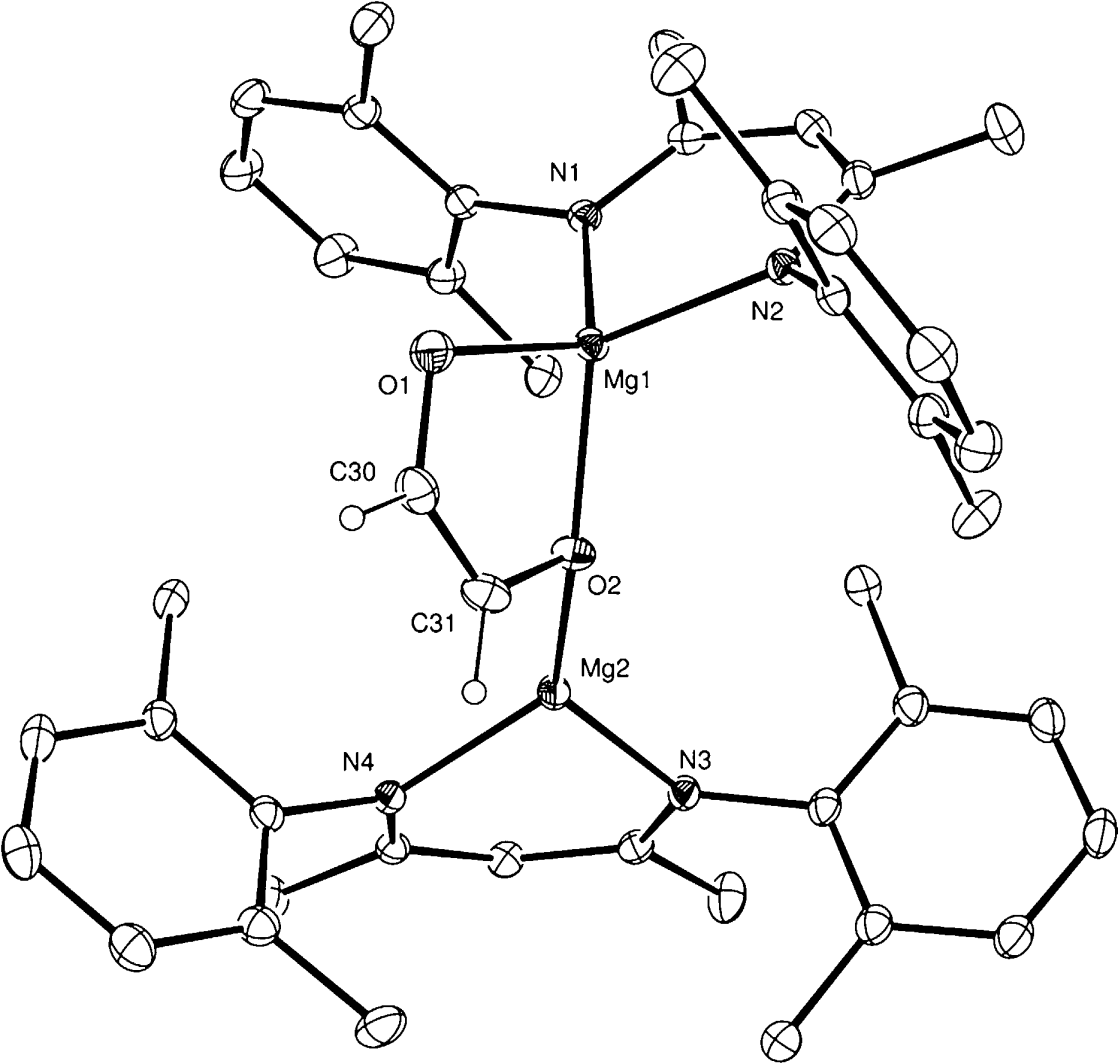
ORTEP representation of compound 1 (25 % probability ellipsoids).^9^ *Iso*-propyl methyl groups and hydrogen atoms except those attached to C30 and C31 are removed for clarity. Selected bond lengths [Å] and angles [°]: Mg1–O1 1.9270(14), Mg1–O2 2.0142(14), Mg1–N1 2.0515(15), Mg1–N2 2.0484(15), Mg2–O2 1.8836(14), Mg2–N4 2.0140(15), Mg2–N3 2.0057(16), C30–C31 1.327(3); O1–Mg1–O2 87.55(6), O1–Mg1–N1 120.14(7), O1–Mg1–N2 118.32(7), O2–Mg2–N3 133.09(7), O2–Mg2–N4 128.81(7), N3–Mg2–N4 97.50(6).

A further reaction between compound **IV** and ^13^CO was performed at −60 °C and monitored by ^1^H and ^13^C NMR spectroscopy. Even under these reduced-temperature conditions, the onset of the formation of compound **1**-^13^C was clearly apparent. An increase in temperature to −40 °C, however, resulted in the observation of a new species (**2**) characterized by a doublet resonance at *δ* 14.08 ppm (^1^*J*_CH_=102.5 Hz) in the ^1^H NMR spectrum (Figure S4) and the appearance of a new, heavily deshielded singlet signal at *δ* 358.9 ppm in the ^13^C{^1^H} spectrum (Figure S5). In the corresponding proton coupled ^13^C NMR spectrum this latter resonance was observed to split into a doublet of doublets (^1^*J*_CH_=102.5, ^2^*J*_HH_=12 Hz; inset, Figure S5), which was shown to be coupled with both the downfield doublet signal in the ^1^H NMR spectrum and a further resonance, otherwise obscured by the β-diketiminate *iso*-propyl methine signal, at about *δ* 2.9 ppm by a heteronuclear multiple bond correlation **(**HMBC) experiment. The low-field ^13^C NMR chemical shift of species **2** is strongly reminiscent of the characteristic acyl carbon resonances observed for a series of thorium(IV) acyl species, [Th(η^5^-C_5_H_5_)_3_(η^2^-COR)] (R=H,3c alkyl^6^), by Marks and co-workers. In these latter cases the dihapto acyl unit was ascribed a modicum of carbene-like character manifest through, in several cases, the observation of a tautomerization process to thorium enolate species. On the basis of these previous observations we ascribe compound **2** as a formyl-hydrido dimagnesium species (see Scheme 1) formed through the reaction of the dimeric compound **IV** with a single molecule of carbon monoxide. No further species could be discriminated during continued monitoring of the low-temperature conversion of **IV** to **1**-^13^C. Although alternative pathways may be envisaged, including the dimerization of two carbene-like formyl units after reaction of **2** with a further equivalent of CO, there is no compelling justification to suggest that the route to compound **1** contrasts with that formerly deduced for the formation of compounds such as **II** and **III**.^3^ We propose, therefore, the reaction pathway shown in Scheme 1 in which the initial carbonylation product, compound **2**, is rapidly consumed through intramolecular hydride transfer to form a dinuclear oxomethylene species **3**. Species **3** is prone to C–C coupling through its reaction with a further molecule of CO while the stereochemistry of the *cis*-enediolate, compound **1**, will ensue through a 1,2-hydrogen shift within species **4**, the selectivity of which is dictated by the anti-periplanar orientation of the C–H bonds to the carbene lone pair across the OCH_2_CO unit.3g

**scheme 1:**
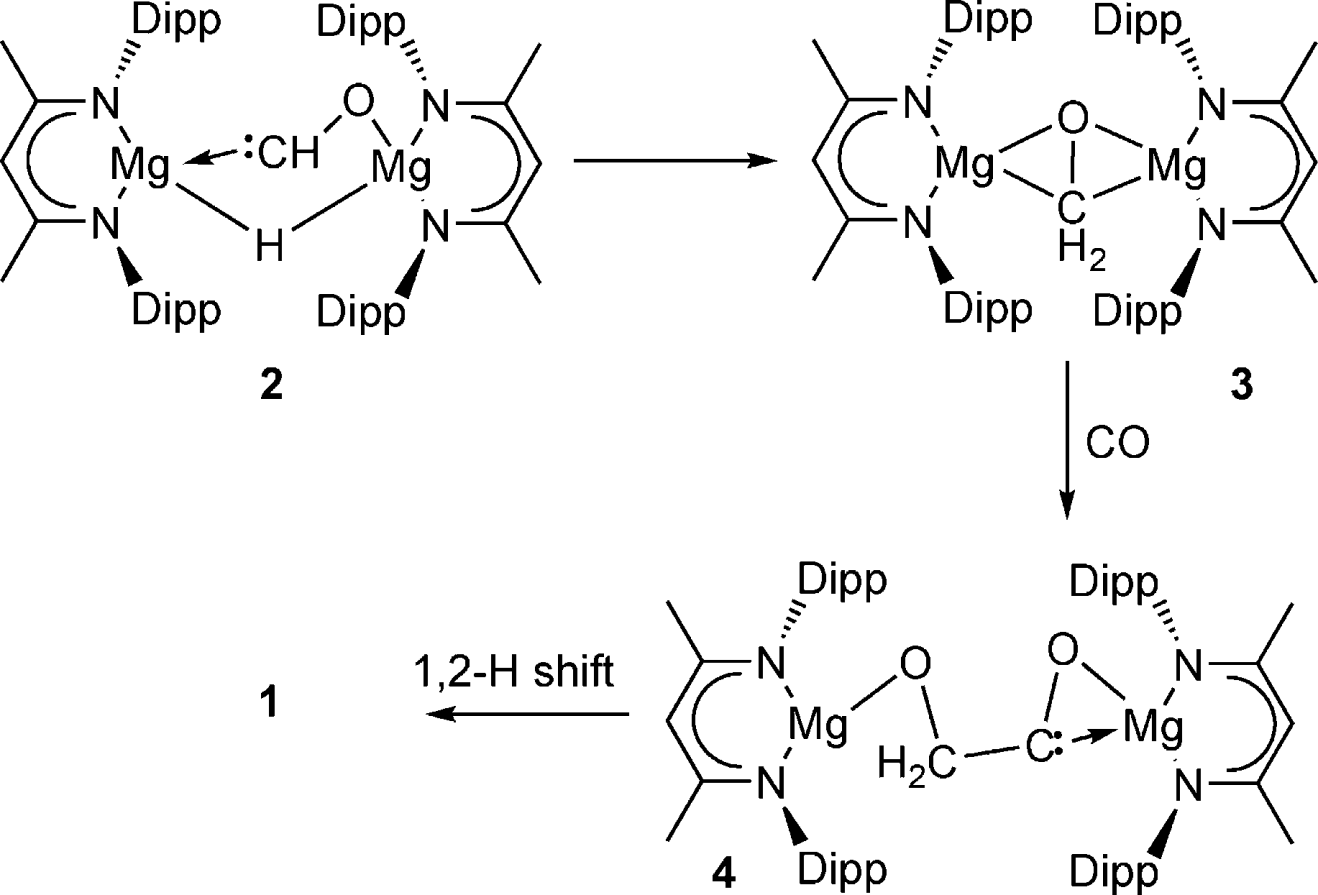
Postulated reaction pathway for the formation of compound 1.

The likely intermediacy of magnesium formyl species during the synthesis of compound **1** was further supported by a study of the reaction of phenylsilane and one atmosphere of ^13^CO in the presence of 10 mol % of **IV**. This reaction proved to be absolutely selective for the deoxygenative reduction of ^13^CO to methylphenylsilane as the ultimate ^13^C-containing product [Eq. (2)].2

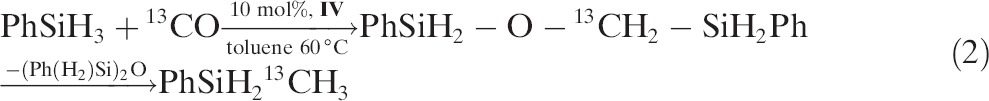
2

The formation of PhSiH_2_^13^CH_3_ was evidenced through the development of a resonance at −7.32 ppm in the ^13^C{^1^H} NMR spectrum, which appeared as a quartet of triplets (^1^*J*_HC_=122.0, ^3^*J*_HC_=5.6 Hz) in the corresponding ^13^C–^1^H gated spectrum (Figures S6 and S7). With no recharging of the ^13^CO in the headspace of the sealed reaction vessel, reactions performed at 60 °C in toluene required 21 days to achieve about 15 % consumption of PhSiH_3_. Although notably slow, these reactions provide the first example of any homogeneous catalytic reduction of CO by a main group system.^7^ Monitoring of the reactions by NMR spectroscopy also revealed the intermediacy of Ph(H_2_)Si–O–CH_2_SiPh(H_2_) (**5**). The methoxysilane species (**5**) was clearly apparent through the observation of a resonance centered at 50.7 ppm in the ^13^C{^1^H} NMR spectrum, which split as a binomial triplet of triplets signal (^1^*J*_HC_=130.7, ^3^*J*_HC_=4.1 Hz) in the ^13^C–^1^H gated spectrum and was consumed simultaneously with the production of PhSiH_2_^13^CH_3_ (Figures S6, S7).

Scheme 2 depicts a provisional reaction mechanism which accounts for these observations. We suggest that the initial step of the catalysis again involves the insertion of CO into the MgH bond of **IV** to form a magnesium formyl similar to species **2**. The absence of any evidence of C_2_ products formed by the reduction of the enediolate component of compound **1**, however, suggests that the metathetical reaction between this formyl species and a Si–H bond of phenylsilane occurs more rapidly than any of the reactivity depicted in Scheme 1. The onward trajectory of the catalysis is then predicated on a sequence of rapid and unobservable C=O/Mg–H and Mg–O/Si–H metathesis events to provide **5**. The rate-determining process of the catalysis is provided by the activation of the C–O bond of compound **5** through O–C/Mg–H and Mg–O/Si–H metathesis steps to yield PhH_2_SiCH_3_ and (PhH_2_Si)_2_O, respectively. The driving force for the reaction is, thus, provided by the production of this latter siloxane by-product.

**scheme 2:**
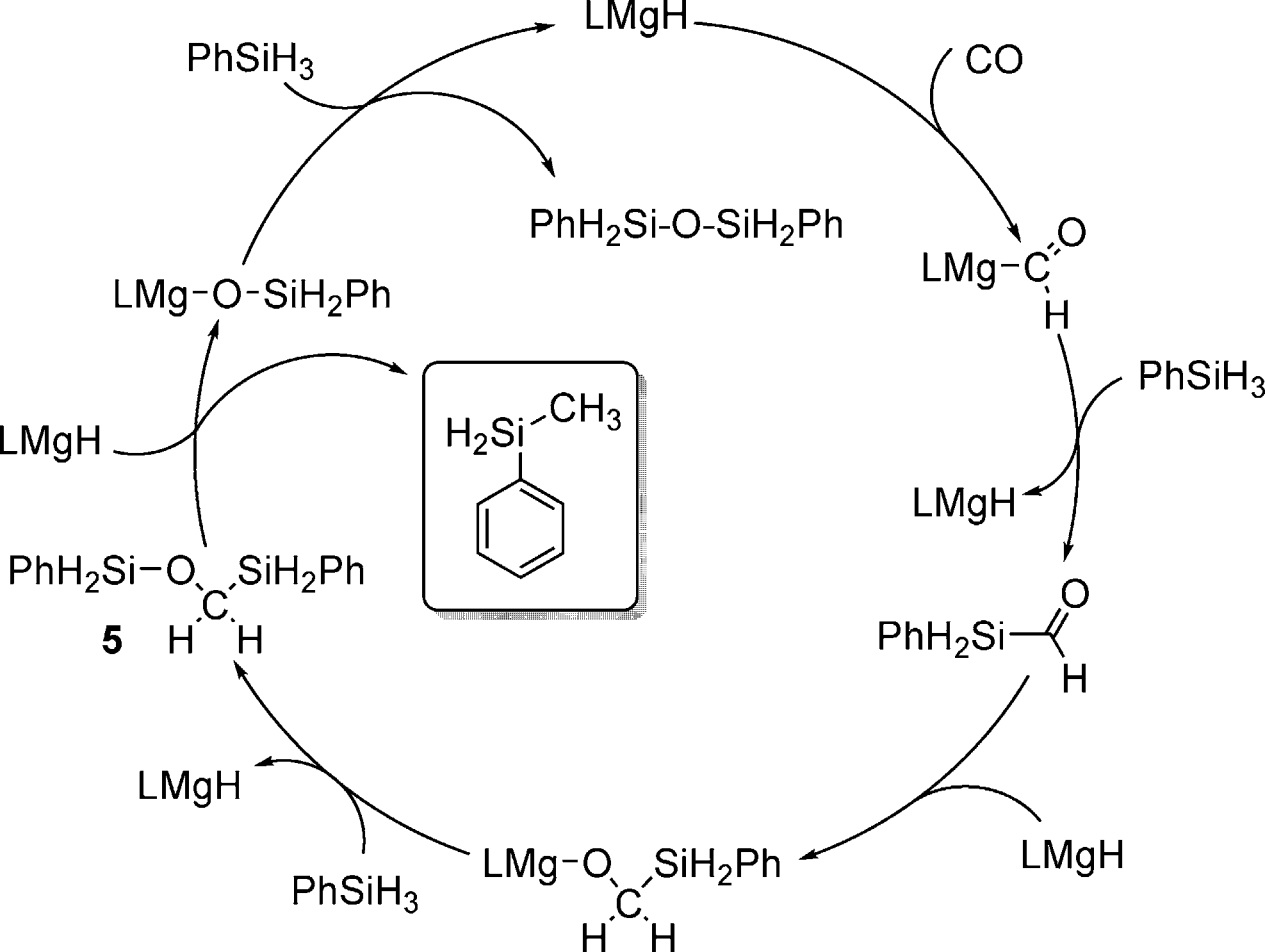
Suggested reaction mechanism for the catalytic reduction of CO with PhSiH_3_ catalyzed by IV.

In conclusion, we have shown that the application of a readily accessible magnesium hydride complex allows the homologation of CO to a *cis*-enediolate species and provides a means to effect its completely selective catalytic deoxygenative reduction under very mild conditions. In further preliminary investigations we have observed that similar but significantly enhanced CO-derived reactivity may be enabled through extensions of this chemistry to heavier Group 2 congeners of magnesium. Application of the conveniently synthesized homoleptic calcium and strontium amides, [Ae{N(SiMe_3_)_2_}_2_] (Ae=Ca, Sr), for example, in catalytic reactions performed under identical conditions to those described above have provided 35 and 40 % conversions, respectively, of the input PhSiH_3_ in only 4 days at 60 °C. We are continuing to explore the mechanistic possibilities presented by this process and to expand the use of CO as both a C_1_ and C_2+_ synthon derived from such highly reactive but earth-abundant alkaline-earth catalysts.

After submission of this manuscript (25/6/15) we became aware of a report of very similar stoichiometric reactivity by Jones and co-workers.^8^ Although an idenitical preparative route to compound **1** is described in this work, the single-crystal X-ray analysis is performed on crystals grown from the coordinating solvent tetrahydrofuran (THF). In **1⋅THF**, therefore, the coordination number of Mg2 is raised to four. A computational density functional theory study of the mechanism of formation of compound **1** is also included in this report. This analysis confirms the viability of the dimeric Mg formyl-hydrido species (**2**) and the gross features of the mechanism shown herein as Scheme 1.

## Supporting Information

As a service to our authors and readers, this journal provides supporting information supplied by the authors. Such materials are peer reviewed and may be re-organized for online delivery, but are not copy-edited or typeset. Technical support issues arising from supporting information (other than missing files) should be addressed to the authors.

miscellaneous_information
