# Supplementary material for: Alkaline-Earth-Promoted CO Homologation and Reductive Catalysis
Source: Angew Chem Int Ed Engl. 2015 Jul 24;54(34):10009–11. doi: 10.1002/anie.201505851 (PMC4678424; doi:10.1002/anie.201505851)
Supplement: Supplementary file 1 [file anie0054-10009-sd1.pdf]

Angewandte Chemie
Eine Zeitschrift der Gesellschaft Deutscher Chemiker

Supporting Information

Alkaline-Earth-Promoted CO Homologation and Reductive Catalysis

Mathew D. Anker, Michael S. Hill, John P. Lowe, and Mary F. Mahon*

anie_201505851_sm_miscellaneous_information.pdf

General experimental procedures

All reactions dealing with air- and moisture-sensitive compounds were carried out under an argon atmosphere using standard Schlenk line and glovebox techniques in an MBraun Labmaster glovebox at O_2, H_2O < 0.1 ppm. NMR experiments using air-sensitive compounds were conducted in J. Youngs tap NMR tubes prepared and sealed in a glovebox under argon. All NMR data were acquired on a Bruker 300 Ultrashield^{TM} for ^{1}H (300 MHz) and ^{13}C{^{1}H} (75.48 MHz) spectra at room temperature or a Bruker 400 Ultrashield^{TM} for ^{1}H (400 MHz) and ^{13}C{^{1}H} (125.76 MHz) spectra. ^{1}H/^{13}C NMR spectra were referenced using residual solvent resonances. Elemental analyses of all moisture- and air-sensitive compounds were performed by Stephen Boyer of London Metropolitan Enterprises. Solvents for air- and moisture-sensitive reactions were provided by an Innovative Technology Solvent Purification System. C_6D_6 and toluene-d_8 were purchased from Fluorochem and dried over molten potassium prior to vacuum transfer into a sealed ampoule and storage in the glovebox under argon. CO was purchased from BOC. Phenylsilane ($PhSiH_3$) and ^{13}CO were purchased from Sigma-Aldrich Ltd. Compound **IV** was synthesized using literature procedures.[1]

Synthesis of compound 1

A solution of compound **IV** (70 mgs, 0.15 mmol) in 0.5 ml toluene was freeze-thaw degassed and exposed to ~1 atm of CO. The reaction flask was resealed and left at room temperature for two hours before the solution was interrogated by NMR spectroscopic analysis, which revealed stoichiometric conversion to compound **1**. The solvent was then removed *in vacuo* and the resultant pale yellow solid was redissolved in *n*-pentane. Storage of this solution at –30ºC for two days afforded crystals of compound **1** suitable for single crystal X-ray diffraction analysis. ^{1}H NMR (d_8-Tol): δ 7.05 – 7.02 (6H, m, Ar-*H*), 5.53 (1H, s, O(*H*C=C*H*)O), 4.79 (1H, s, C-*H*), 3.14 (4H, m., $^{3}J_{HH}$ = 6.85 Hz, C*H*(CH_3)$_2$), 1.56 (6H, s, NC(C*H*$_3$)CH), 1.17 (12H d, $^{3}J_{HH}$ = 6.85 Hz, CH(C*H*$_3$)$_2$), 1.02 (12H d, $^{3}J_{HH}$ = 6.85 Hz, CH(C*H*$_3$)$_2$). ^{13}C{^{1}H} NMR (d_8-Tol, 125.76 MHz): δ 170.0 (N*C*(CH_3)CH), 144.6 (*ipso*-Ar), 142.5 (*o*-Ar), 131.9 (O(H*C*=*C*H)O), 125.8 (*p*-Ar), 123.8 (*m*-Ar), 95.5 (NC(CH_3)*C*H), 28.6 (N*C*H(CH_3)$_2$), 24.7, 24.2(CH(*C*H$_3$)$_2$). Anal. Calcd. for $C_{60}H_{84}Mg_2N_4O_2.2H_2O$: C, 73.74; H, 9.05; N, 5.72%. Found: C, 73.88; H, 9.14; N, 7.71%. The sample was highly hydroscopic and sequestered two water molecules on exposure to atmosphere.

Figure S1: The AA'XX' spin system observed in the gated ^{13}C-^{1}H NMR spectrum of compound **1**.

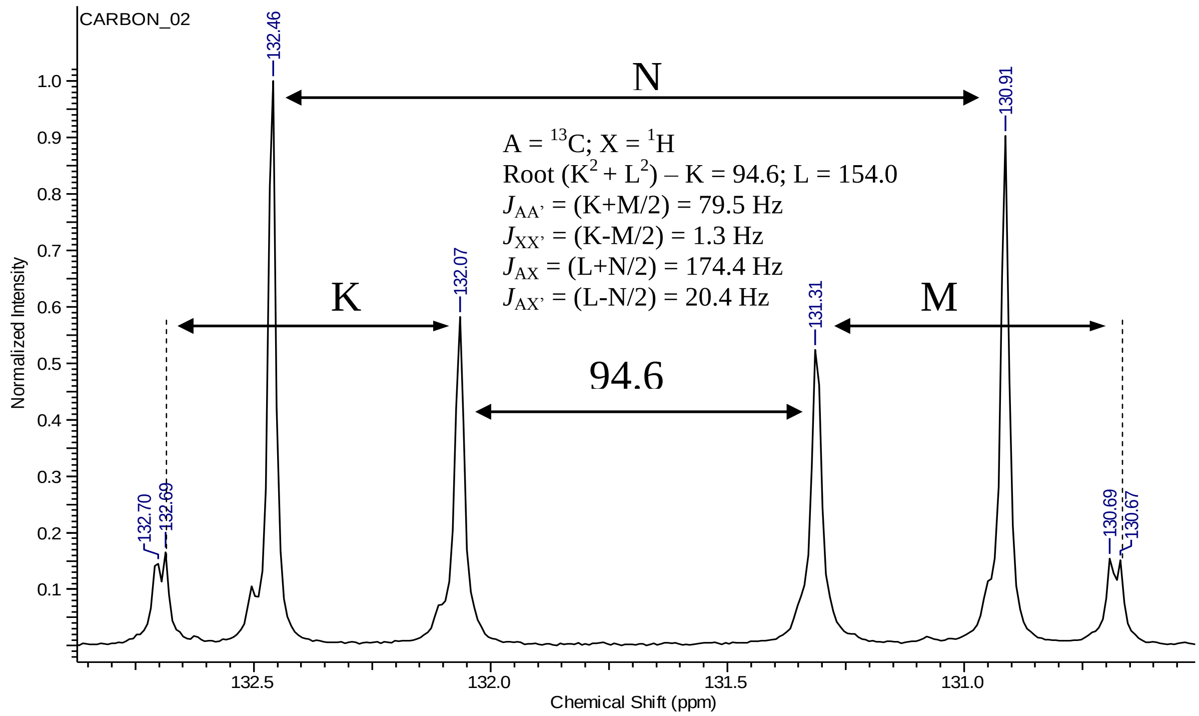

Figure S2: Simulations of the AA'XX' spin systems observed in (a) the ^{1}H NMR and (b) the gated ^{13}C-^{1}H NMR spectra of compound **1**.

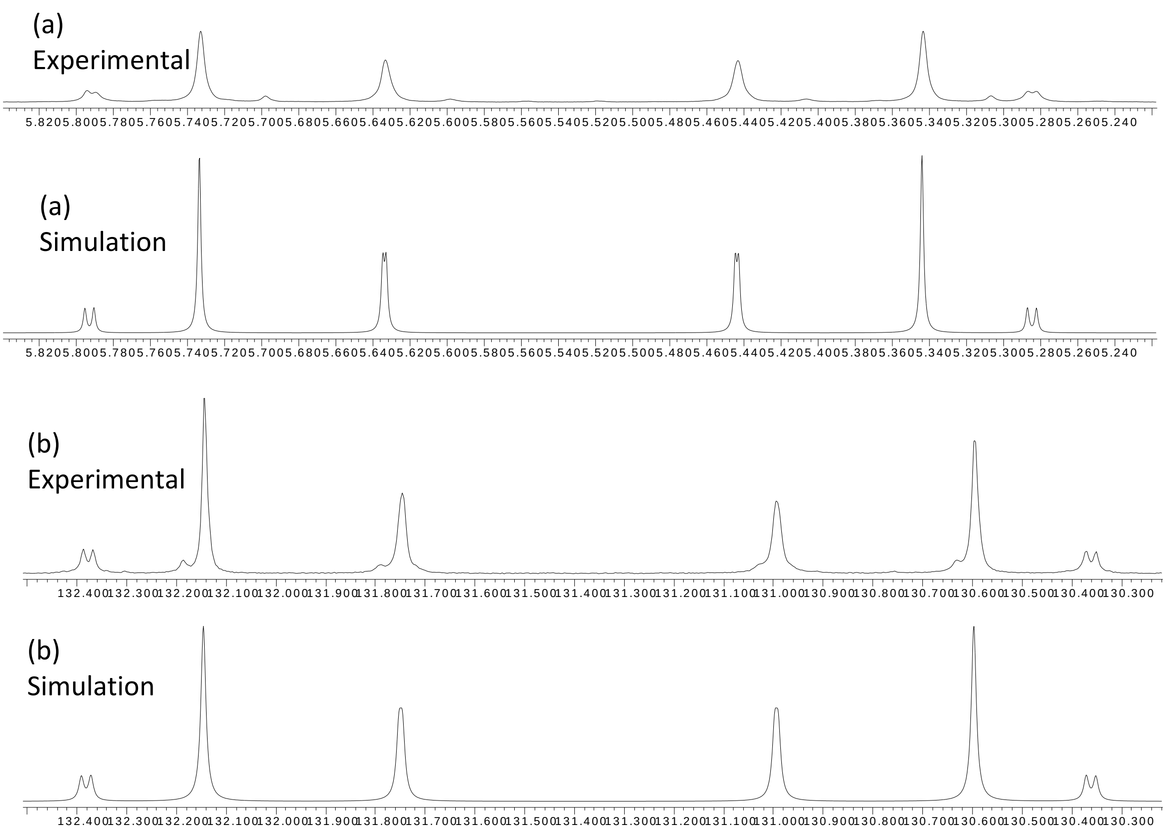

Figure S3: VT ^{1}H NMR spectra of the β-diketiminate and enediolate methine region of compound **1**.

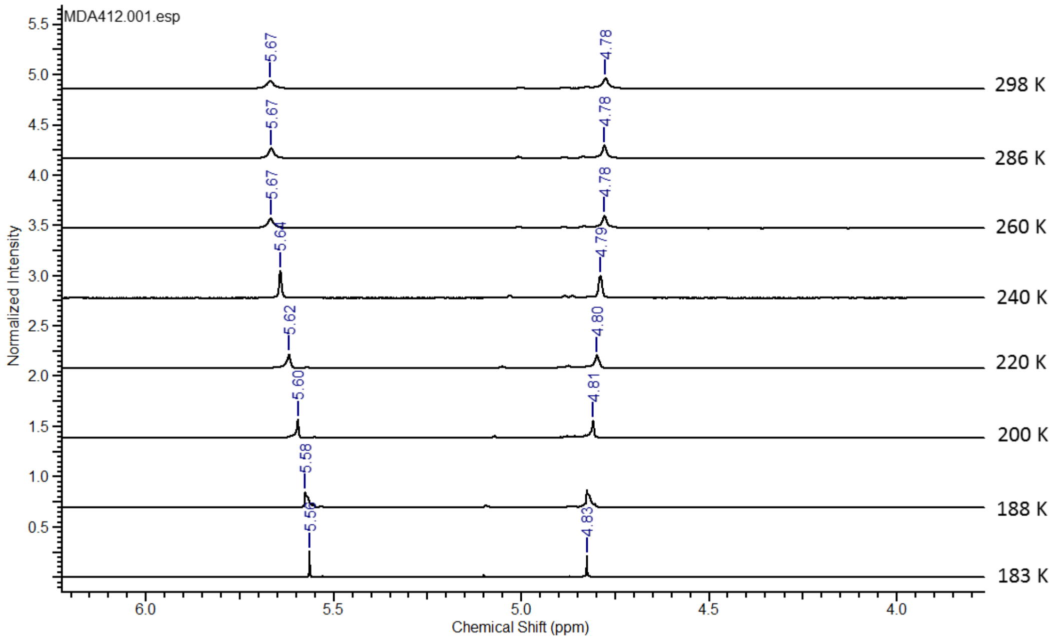

Figure S4: Formyl signal observed in the ^{1}H NMR spectrum of a reaction performed between compound **IV** and ^{13}CO at –40ºC.

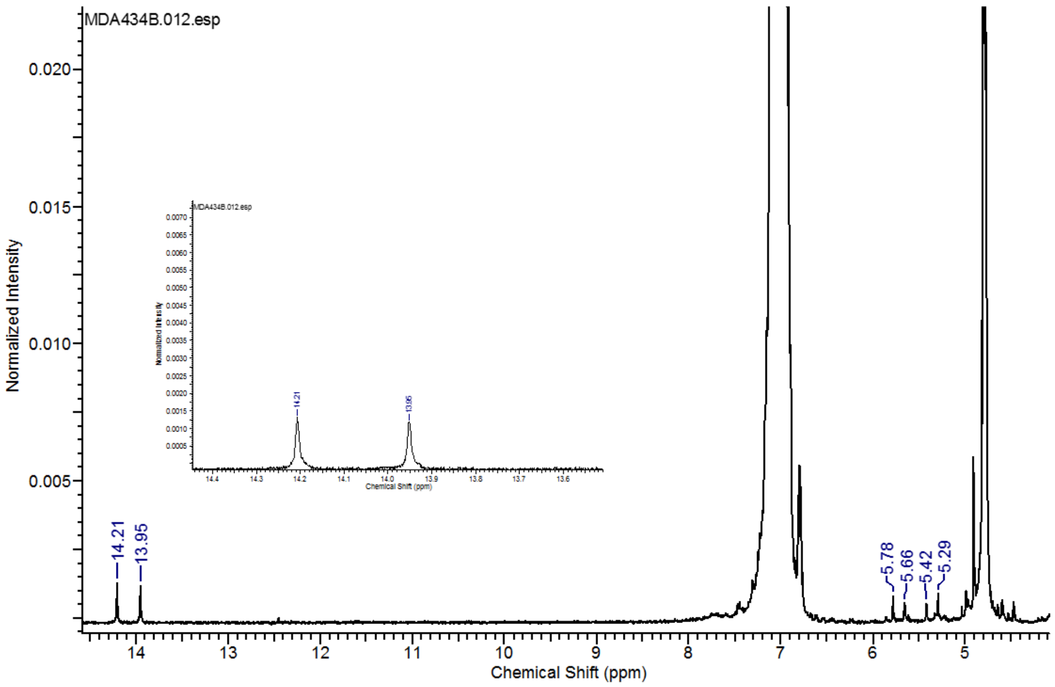

Figure S5: Formyl signal observed in the gated ^{13}C-^{1}H NMR spectrum of a reaction performed between compound **IV** and ^{13}CO −40ºC.

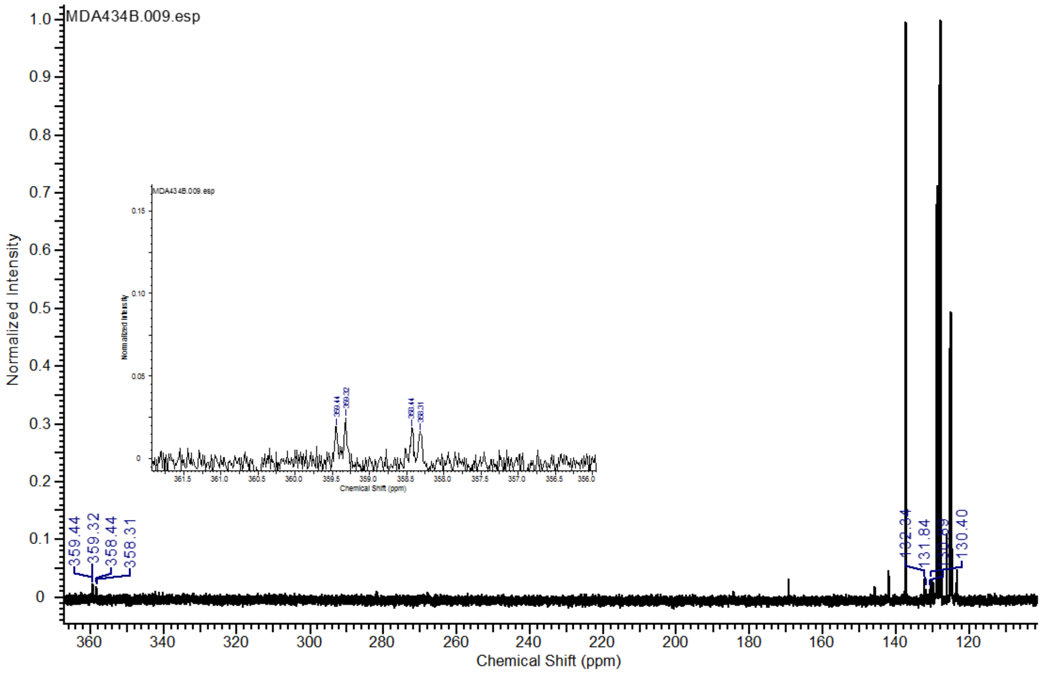

X-ray analysis of compound 1

Single crystals of $C_{60}H_{84}Mg_2N_4O_2$, compound **1**, were obtained from *n*-pentane solution at –30ºC. A suitable crystal was selected and data were collected on a SuperNova, Dual, Cu at zero, EosS2 diffractometer. The crystal was kept at 150(2) K during data collection. Using Olex2,[2] the structure was solved with the olex2.solve[3] structure solution program using Charge Flipping and refined with the ShelXL[4] refinement package using Least Squares minimization.

Crystal Data for $C_{60}H_{84}Mg_2N_4O_2$ (M =941.93 g mol^{-1}): monoclinic, space group $P2_1/n$ (no. 14), a = 13.3888(2) Å, b = 29.4751(4) Å, c = 14.56423(18) Å, β = 93.8392(12)°, V = 5734.67(13) Å^3, Z = 4, T = 150(2) K, μ(CuKα) = 0.695 mm^{-1}, D_c = 1.091 g/cm^3, 22315 reflections measured (8.546° ≤ 2θ ≤ 139.702°), 10569 unique (R_{int} = 0.0423, R_{sigma} = 0.0545) which were used in all calculations. The final R_1 was 0.0482 ($I > 2\sigma(I)$) and wR_2 was 0.1249 (all data).

Catalytic reduction of CO (representative procedure)

In a glovebox, to a vial containing compound **IV** (0.02 mmol, 10 mg), was added toluene-d_8 (0.5 mL) followed by $PhSiH_3$ (0.2 mmol, 27.8µl). The resultant solution was transferred to a NMR tube equipped with a J. Youngs tap which was sealed and removed from the glovebox. The solution was then freeze pumped thaw degassed to remove argon, exposed to 1 atmosphere of CO, resealed and warmed to 60°C. NMR analysis was performed at regular intervals and conversions were analyzed by ratios of starting material to product, with products identified by comparison to literature values.

Figure S6: $^{13}C\{^1H\}$ NMR spectra recorded after (a) one day, (b) three days, (c) one week during the reaction of $PhSiH_3$ and ^{13}CO catalyzed by 10 mol% **IV** at 60°C.

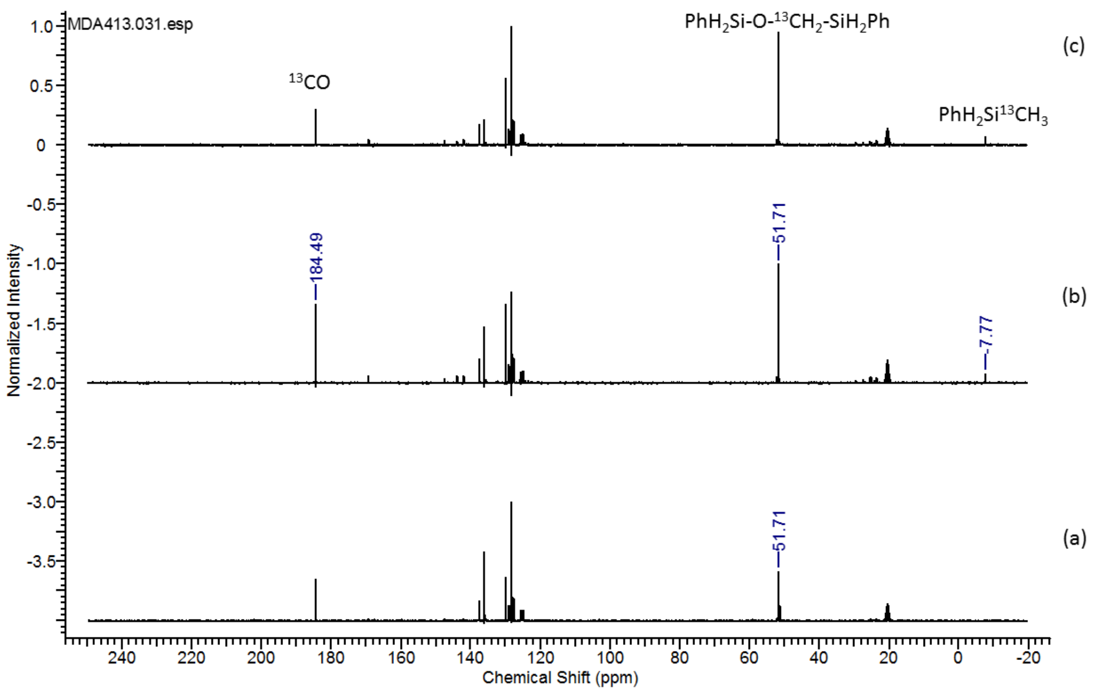

Figure S7: Expansions of the signals assigned to (a) $PhH_2Si\text{-}O\text{-}^{13}\underline{C}H_2\text{-}SiH_2Ph$ and (b) $PhH_2Si\text{-}^{13}\underline{C}H_3$ products in the gated $^{13}C\text{-}^1H$ NMR spectra during the reaction of $PhSiH_3$ and ^{13}CO catalyzed by 10 mol% **IV** at 60°C.

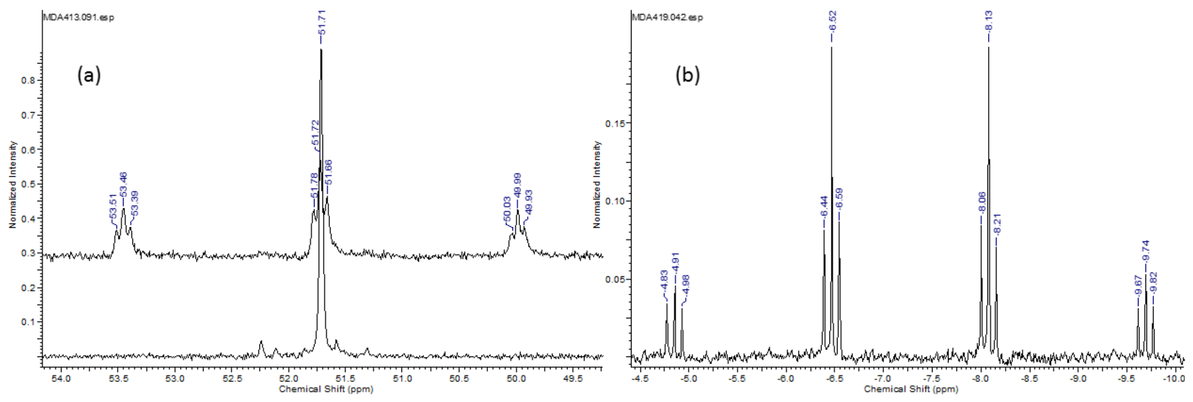

References

[1] S. J, Bonyhady, C. Jones, S. Nembenna, A. Stasch, A. J. Edwards, G. J. McIntyre, *Chem. Eur. J.* **2010**, *16*, 938-955.

[2] O. V. Dolomanov, L. J. Bourhis, R. J. Gildea, J. A. K. Howard, H. Puschmann, *J. Appl. Cryst*. **2009**, *42*, 339-341.

[3] L. J. Bourhis, O. V. Dolomanov, R. J. Gildea, J. A. K. Howard, H. Puschmann, *Acta Cryst. A* **2015**, *71*, 59-75.

[4] G. M. Sheldrick, *Acta Cryst. A*, **2008**, *64*, 112-122.
